# Lipocalin-2 Serum Levels in Rheumatoid Arthritis Patients Treated with Adalimumab and Its Correlation with Proinflammatory Factors

**DOI:** 10.1155/2024/7264704

**Published:** 2024-10-07

**Authors:** Javier Conde-Aranda, Morena Scotece, María Varela-García, Carlos Torrijos-Pulpón, Laura Arosa, Miguel Camba-Gómez, Jesús Pino, Oreste Gualillo

**Affiliations:** ^1^Molecular and Cellular Gastroenterology, Health Research Institute of Santiago de Compostela (IDIS), Santiago de Compostela, Spain; ^2^SERGAS (Health Service from Galicia) and IDIS (Health Research Institute of Santiago de Compostela), The NEIRID Lab (Neuroendocrine Interactions in Rheumatology and Inflammatory Diseases), Research Laboratory 9, Santiago University Clinical Hospital, Santiago de Compostela, Spain; ^3^SERGAS (Health Service from Galicia), Division of Orthopaedics Surgery and Traumatology, Santiago University Clinical Hospital, Santiago de Compostela, Spain; ^4^Department of Surgery and Medical-Surgery Specialities, University of Santiago de Compostela, Santiago de Compostela, Spain

## Abstract

**Background:**

Obesity is associated with an increased risk for different chronic diseases such as osteoarthritis (OA) or rheumatoid arthritis (RA). In fact, adipose tissue is now recognized as an endocrine organ able to secrete a wide variety of factors called adipokines, which have been demonstrated to participate in the pathophysiology of RA by regulating inflammation and immunity. LCN2 is one of these adipose tissue-derived factors. However, scarce information is available about the levels of this adipokine in different rheumatic diseases. Therefore, we aimed to analyze LCN2 serum levels in healthy, OA, and RA patients under different treatments.

**Methods:**

Serum levels of LCN2, among other proinflammatory and chemotactic factors, have been measured by ELISA or Multiplex in the following four groups of individuals: healthy, OA, and RA patients treated with conventional treatment or adalimumab.

**Results:**

We found increased serum levels of LCN2 in OA and RA patients. Interestingly, LCN2 serum levels show a similar pattern to that observed for different proinflammatory and chemotactic factors, being increased in RA conventional treated patients in comparison to RA patients treated with adalimumab. Also, RA patients under conventional treatment revealed a positive and significant correlation between LCN2 and CCL2, CCL3, IL-8, IL-1*β*, IL-6, and CRP. In patients with RA treated with adalimumab, only IL-6 and CRP correlated significantly with LCN2.

**Conclusions:**

Our results clearly suggest that LCN2 is modulated and associated with inflammation in rheumatic diseases. Therefore, the serum levels of this adipokine might be used as an additional biomarker of the inflammatory/disease activity.

## 1. Introduction

Rheumatoid arthritis (RA) is a chronic inflammatory autoimmune disease, characterized by persistent synovitis that leads to joint tissue destruction, resulting in impaired articulation function. Abnormal cellular and humoral immune responses lead to the presence of autoantibodies and the infiltration of immune cells into the synovium. At this point, the excessive production of proinflammatory cytokines perpetuates the inflammatory response and the damage of joint tissues [1, 2].

RA is a disease with unknown etiology, although some environmental factors, such as tobacco smoke, have been identified as a risk factor for developing RA [3]. Obesity is a worldwide pandemic, and it has been also associated with increased risk for different chronic diseases. In fact, it is feasible to think of the existence of a relationship between obesity and RA, since adipose tissue inflammation may be linked with systemic chronic inflammation [4]. Moreover, adipose tissue is now recognized as an endocrine organ able to secrete a wide variety of factors called adipokines, which have been demonstrated to participate in the pathophysiology of RA by regulating inflammation and immunity [5, 6, 7, 8].

Lipocalin-2 (LCN2), also known as siderocalin, 24p3, uterocalin, and neutrophil gelatinase-associated lipocalin (NGAL), is a 25-kDa glycoprotein isolated from neutrophil granules, although white adipose tissue is thought to be another main source [9, 10]. The LCN2 protein has been isolated as a 25-kDa monomer, as a 46-kDa homodimer, and in a covalent complex with matrix metalloproteinase-9 (MMP9). This adipokine participates in different physiological and pathological processes such as the induction of apoptosis in hematopoietic cells [11], transport of fatty acids and iron [12, 13], modulation of inflammation [14], metabolic homeostasis [15], regulation of iron metabolism [16], and suppression of bacterial growth [17].

At present, some evidence suggests the participation of LCN2 in the development of different rheumatic diseases. Our group and others demonstrated the expression of this adipokine in chondrocytes and its induction by different proinflammatory stimuli [18, 19, 20]. Urinary LCN2 levels were higher in systemic lupus erythematosus (SLE) patients compared to healthy controls [21]. Interestingly, SLE patients with lupus nephritis presented increased urinary LCN2 concentrations compared to those without nephritis [21], suggesting that this adipokine may be a potential marker of the severity of renal involvement in patients with SLE [22, 23]. In line with this, it was reported that synovial fluid from RA patients had higher levels of LCN2 in comparison to synovial fluid obtained from osteoarthritis (OA) patients [24]. Moreover, increased LCN2 serum levels were found to be higher in RA patients in comparison to controls [25]. Also, studies in chondrocytes [20] and osteoblasts [26] demonstrated the implication of LCN2 in OA pathophysiology. Interestingly, LCN2 contributes to the increase of immune/inflammatory environment in OA patients through the induction of proinflammatory factors such as IL-8, IL-6, and MIP-1*α* in CD4+ T cells from OA patients [27]. Therefore, there is a substantial amount of information suggesting the participation/association of LCN2 with musculoskeletal diseases. However, despite this foregoing data, the serum levels of LCN2 in OA patients have not been explored yet. It is not known either the circulating levels of this adipokine correlate with the inflammatory status in patients with RA under conventional or biologic therapy. To tackle those knowledge gaps, we analyzed the serum levels of LCN2 in OA patients and RA patients subjected to conventional therapy or treated with adalimumab. For completeness, we also investigated the correlation between LCN2 and different proinflammatory and chemotactic factors.

## 2. Materials and Methods

### 2.1. Patients and Samples

Serum from 30 RA patients with conventional treatment (methotrexate and prednisone) and 35 patients with adalimumab treatment classified according to the 1987 American College of Rheumatology criteria were included in this study. Also, 20 nonmatched healthy subjects and 35 patients with OA were enrolled in this study. Measurements of height and weight were performed in order to calculate the body mass index (BMI). To see other data from subjects, please see Table 1. All blood samples were collected, aliquoted, and frozen at −80°C.

This study was approved by the local ethics committee (Comité de Investigación de Santiago-Lugo) (Cod. 2014/310), and all participants signed their informed consent. Patients with autoimmune and/or chronic malignancies, infections, or metabolic diseases were excluded from the study.

### 2.2. Enzyme-Linked Immunosorbent Assay

We analyzed the serum levels of LCN2 of healthy subjects, OA and RA patients by using the human lipocalin-2/NGAL ELISA from BioVendor (Karasek, Czech Republic), following the manufacturer's instructions. We also measured the levels of C-reactive protein (CRP) by using the Human CRP ELISA Kit from Raybiotech (GA, USA).

### 2.3. Multiplex System

We analyzed the serum levels of TNF-*α*, IL-8, IL-1*β*, CCL2, CCL3, and IL-6 of healthy and RA subjects by using Bio-Plex Multiplex System from Bio-Rad (CA, USA), following the manufacturer's instructions.

### 2.4. Statistical Analysis

Data are reported as mean ± standard error (SEM) (error bars) of the independent samples. Statistical analyses were performed by ANOVA followed by unpaired *t*-test and Student–Newman–Keuls test, using the GraphPad Prism 8 software, with *p* values < 0.05 considered significant. Correlations were performed using the Spearman's rank correlation coefficient (*r*).

## 3. Results

### 3.1. LCN2 Serum Levels Are Increased in RA and OA Patients

**Figure 1:**
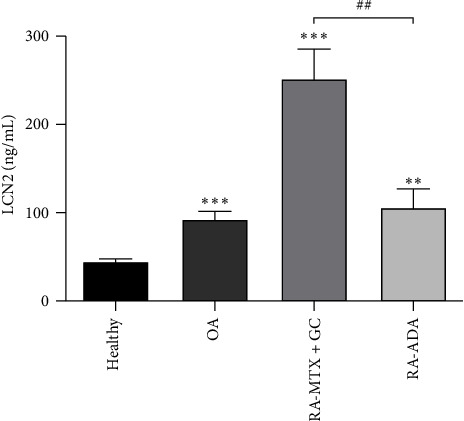
LCN2 protein level determination by ELISA in the serum of the indicated groups. The data shown represent mean ± SEM.  ^*∗∗*^*P* ≤ 0.01;  ^*∗∗∗*^*P* ≤ 0.001 relative to healthy. ##*P* ≤ 0.01 relative to RA-MTX+GC.

By ELISA assay, we analyzed LCN2 serum levels in four different groups, including healthy subjects, OA patients, RA patients with conventional treatment, and RA patients treated with adalimumab. As shown in Table 1 and Figure 1(a), LCN2 serum levels were significantly increased in all groups analyzed in comparison to the healthy subjects group, showing the RA group with conventional treatment the highest serum levels of LCN2. Interestingly, the RA–adalimumab group presented a strong and significant decrease in LCN2 levels as compared to the RA–conventional group (Figure 1(a)).

### 3.2. RA Patients Show a Similar Profile in the Levels of Inflammation and Chemotactic Markers as that Observed for LCN2

**Figure 2:**
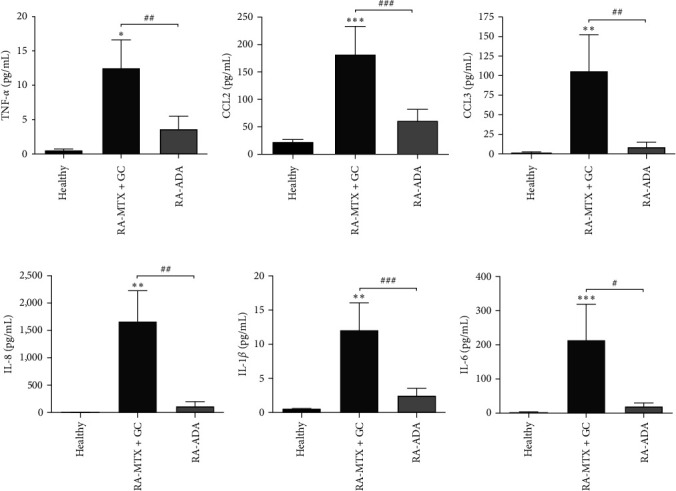
TNF-*α*, CCL2, CCL3, IL-8, IL-1*β*, and IL-6 protein level determination by Multiplex in the serum of the indicated groups. Data shown in (a), (b), (c), (d), (e), and (f) represent mean ± SEM.  ^*∗*^*P* ≤ 0.05;  ^*∗∗*^*P* ≤ 0.01;  ^*∗∗∗*^*P* ≤ 0.001 relative to healthy. #*P* ≤ 0.05; ##*P* ≤ 0.01; ###*P* ≤ 0.001 relative to RA-MTX + GC.

To investigate the circulating levels of other markers implicated in RA pathophysiology, we performed a Bio-Plex multiplex assay of several cytokines such as TNF-*α*, IL-1*β*, and IL-6 and chemokines CCL2, CCL3, and IL-8. As expected, the RA–adalimumab group presented decreased serum levels of TNF-*α* in comparison to the RA–conventional group (Figure 2(a)). Interestingly, we observed a significant decrease in all the factors analyzed in the RA–adalimumab group compared with the RA–conventional group (Figures 2(b), 2(c), 2(d), 2(e), and 2(f)).

### 3.3. LCN2 Positively Correlates with Markers of Inflammation and Chemotaxis

RA is characterized by a prominent immune and inflammatory response which can be modulated by anti-TNF therapy. As described above, LCN2 and other proinflammatory and chemotactic factors were regulated by adalimumab treatment; therefore, we sought to ascertain, using the Spearman correlation test, whether LCN2 is related to those markers in RA patients.

We first analyzed the relationship between LCN2 and TNF-*α* serum levels in the RA–conventional group. Although there is a positive trend between these two factors, we found the correlation not statistically significant (Table 2). However, we observed a positive and significant correlation between LCN2 and different proinflammatory markers such as IL-1*β*, IL-6, and CRP (Table 2). Moreover, we detected that LCN2 serum levels were positively correlated with the chemokines CCL2, CCL3, and IL-8 (Table 2). We also investigated the existence of a relationship between LCN2 and BMI, but we did not detect a significant correlation between these two parameters. Interestingly, in the RA–adalimumab group, among all the factors tested, we found that LCN2 only correlated positively with IL-6 and CRP (Table 3).

To note, we did not detect any correlation between LCN2 and CRP levels in both healthy and OA groups (data not shown).

## 4. Discussion

Obesity has been recently considered an increased risk factor for developing RA. Actually, it is generally accepted that the levels of certain adipokines, such as leptin, are increased in RA [5, 28]. In addition, it was postulated that adipokines' concentration in serum or synovial fluid could be associated with joint damage or disease activity in RA or OA [5, 29, 30]. All these data suggest that adipokines participate in the development and/or progression of different rheumatic diseases, and the study of the role played by these factors could be useful for searching new therapeutic approaches or biomarkers for a better diagnosis.

In this study, we aimed to investigate LCN2 serum levels in OA and RA patients who underwent to different treatments to ascertain whether this adipokine is influenced by the inflammatory status. Indeed, and in agreement with previously reported results by Katano et al. using synovial fluid from RA and OA patients [24], we also observed higher serum levels of LCN2 in RA patients treated with conventional therapy, with a worse inflammatory profile, in comparison to the OA group. To note, LCN2 circulating concentrations in OA patients are even significantly increased when compared to healthy controls, suggesting that articular alterations involving any degree of inflammation are enough to modulate the production of this adipokine, and therefore, LCN2 could work as a marker of joint damage. In fact, we confirmed the elevated LCN2 serum levels that were recently described in RA patients versus healthy subjects [25], but we showed for the first time a dramatic decrease of LCN2 serum levels in RA patients responding to adalimumab as compared to RA patients under conventional treatment. These findings highlight that LCN2 is closely linked to the disease activity. Therefore, we performed additional correlation analysis to confirm that hypothesis. Our results clearly demonstrate that LCN2 serum levels are modulated by inflammatory/immune state, since RA patients with conventional treatment, presenting a worse proinflammatory cytokine/chemokine profile, showed the highest levels of this adipokine. Actually, our data demonstrated that LCN2 correlated positively with different proinflammatory markers and chemokines. Although LCN2 was postulated as a metabolic syndrome-related protein, we did not observe any correlation between this protein and BMI in RA patients. Notably, the fact that LCN2 was positively correlated with CRP suggests that this protein is likely more related to inflammation than metabolic status. This result agrees with those observed in psoriatic patients [31] or subjects with metabolic syndrome [32]. Similarly, in RA patients undergoing infliximab treatment, a correlation between resistin and CRP was also observed [33]. Nonetheless, no correlation was found between this adipokine and different metabolic syndrome parameters in those patients [33], suggesting that LCN2 and resistin might be better markers of inflammation rather than metabolic dysbalance. This is in contradiction to adiponectin levels in RA patients with high-grade inflammation, in which low adiponectin levels clustered with features of metabolic syndrome that contribute to cardiovascular complications in RA [34]. Cardiovascular risk assessment is crucial since it is the most common cause of premature mortality in patients with RA [35]. A recently published meta-analysis has shown that elevated LCN2 was linked to major adverse cardiovascular events in acute coronary syndrome patients [36]. Therefore, future studies including detailed associations between LCN2 and metabolic or other cardiovascular disease risk parameters will be relevant to determine the use of this adipokine in the context of RA.

The use of LCN2 as a biomarker for other immune-mediated diseases was postulated. SLE patients with lupus nephritis presented increased urinary LCN2 concentrations compared to those without nephritis [21]. In addition, LCN2 levels were increased in patients with sepsis and other infections and in patients with different inflammatory diseases [31, 37, 38]. Notably, this adipokine was strongly associated with different parameters of inflammation [31, 39], which suggests a role for LCN2 as a biomarker of inflammatory status. Then, it is feasible to think that LCN2 could be also used in RA patients as an inflammatory and/or disease activity marker.

## 5. Conclusions

In summary, we showed for the first time that OA patients had increased serum levels of LCN2 compared with healthy subjects. In RA patients, we found higher serum levels of LCN2 in the conventional treatment group compared with patients responding to adalimumab. Overall, our results clearly suggest that LCN2 is modulated and associated with joint inflammation. Therefore, we open the possibility of using LCN2 serum levels as a biomarker of the inflammatory/disease activity in rheumatic diseases.

## Figures and Tables

**Table 1:** Anthropometric and indicated protein levels in the different studied groups.

Characteristics	Healthy subjects	Osteoarthritis patients	RA patients (conventional therapy)	RA patients (adalimumab)
	(*n* = 20)	(*n* = 35)	(*n* = 30)	(*n* = 35)
Age (years; median)	30.1	69.4	46.4	57.2
Sex (*n* (female/male))	11/9	22/13	27/3	32/3
BMI (kg/m^2^)	23.03	29.3	29.8	27.1
LCN2 (ng/mL)	44.67 ± 3.167	92.66 ± 8.944	251.8 ± 33.84	106 ± 21.10
CRP ng/ml	2,920 ± 681.9	11,065 ± 2263	22,016 ± 5375	12,433 ± 3424

**Table 2:** Correlations of the indicated proteins in the RA-MTX+GC group.

Characteristics	Correlation	RA conventional therapy
		Lipocalin-2 (LCN2) in serum levels
		(ng/mL)
BMI	*r*	0.2212
(kg/m^2^)	*p*	0.2402
TNF*α*	*r*	0.3141
(pg/mL)	*p*	0.0971
IL1*β*	*r*	0.6561
(pg/mL)	*p*	0.0001
IL-6	*r*	0.6176
(pg/mL)	*p*	0.0050
CCL2	*r*	0.3966
(pg/mL)	*p*	0.0332
CCL3	*r*	0.7186
(pg/mL)	*p*	0.0001
IL-8	*r*	0.7818
(pg/mL)	*p*	0.0001
CRP	*r*	0.3715
(ng/mL)	*p*	0.0433

**Table 3:** Correlations of the indicated proteins in the RA-ADA group.

Characteristics	Correlation	RA adalimumab
		Lipocalin-2 (LCN2) in serum levels
		(ng/mL)
BMI	*r*	0.04105
(kg/m^2^)	*p*	0.8357
TNF*α*	*r*	0.1558
(pg/mL)	*p*	0.4287
IL1*β*	*r*	0.3920
(pg/mL)	*p*	0.0643
IL-6	*r*	0.4701
(pg/mL)	*p*	0.0236
CCL2	*r*	0.2065
(pg/mL)	*p*	0.3444
CCL3	*r*	0.3719
(pg/mL)	*p*	0.0806
IL-8	*r*	0.1794
(pg/mL)	*p*	0.4128
CRP	*r*	0.5316
(ng/mL)	*p*	0.0015

## Data Availability

Data obtained from ELISA or Multiplex are available from the corresponding authors on reasonable request.
